# Mechanical Properties of Optimized Diamond Lattice Structure for Bone Scaffolds Fabricated via Selective Laser Melting

**DOI:** 10.3390/ma11030374

**Published:** 2018-03-03

**Authors:** Fei Liu, David Z. Zhang, Peng Zhang, Miao Zhao, Salman Jafar

**Affiliations:** 1 State Key Laboratory of Mechanical Transmission, Chongqing University, Chongqing 400044, China; liufei29@cqu.edu.cn (F.L.); pengzhang@cqu.edu.cn (P.Z.); zhaomiaocqu@gmail.com (M.Z.); salman6537@gmail.com (S.J.); 2 College of Engineering, Mathematics and Physical Sciences, University of Exeter, North Park Road, Exeter EX4 4QF, UK

**Keywords:** lattice structure, structure optimization, bone scaffolds, compressive deformation behavior, selective laser melting, laser powder bed fusion

## Abstract

Developments in selective laser melting (SLM) have enabled the fabrication of periodic cellular lattice structures characterized by suitable properties matching the bone tissue well and by fluid permeability from interconnected structures. These multifunctional performances are significantly affected by cell topology and constitutive properties of applied materials. In this respect, a diamond unit cell was designed in particular volume fractions corresponding to the host bone tissue and optimized with a smooth surface at nodes leading to fewer stress concentrations. There were 33 porous titanium samples with different volume fractions, from 1.28 to 18.6%, manufactured using SLM. All of them were performed under compressive load to determine the deformation and failure mechanisms, accompanied by an in-situ approach using digital image correlation (DIC) to reveal stress–strain evolution. The results showed that lattice structures manufactured by SLM exhibited comparable properties to those of trabecular bone, avoiding the effects of stress-shielding and increasing longevity of implants. The curvature of optimized surface can play a role in regulating the relationship between density and mechanical properties. Owing to the release of stress concentration from optimized surface, the failure mechanism of porous titanium has been changed from the pattern of bottom-up collapse by layer (or cell row) to that of the diagonal (45°) shear band, resulting in the significant enhancement of the structural strength.

## 1. Introduction

Recently, cellular lattice structures featuring multifunctional performances [1] including high strength, lightweight and good energy absorption have been extensively studied as a suitable candidate for biomedical applications such as osseointegration or bone grafting [2,3] due to their inner topological complexity and comparable properties to the host bone. In such applications, a biomaterial should be biocompatible, not only possessing similar structural and mechanical properties to that of the bone it replaces, especially Young’s modulus, but also retaining biological activities for tissue ingrowth and optimal osseointegration [4,5].

Lattice structures characterized by non-stochastic open unit cells have better mechanical properties in comparison to stochastic foams that exhibit localized deformations from internal imperfections [5,6,7]. Their controllable morphological parameters, such as pore architecture, pore size and volume fraction, can tailor the biomechanical properties matching the host tissue well, and the permeability from interconnected structures facilitates cell migration and vascularization to stimulate bone ingrowth [8,9]. For example, by adjusting the morphology of structures, the elastic modulus and porosity of the cellular lattices can be custom-designed to the levels of replaced bones for reducing stress-shielding effects [10]. Melchels et al. [11] assessed the influence of scaffold pore architecture on cell seeding and static culturing. It was shown that the gyroid-type lattice structure has a more than 10-fold higher permeability than that of salt leaching architecture due to pore interconnections, promoting homogeneous cell distribution and improving cell seedability. In addition, sprayed with hydroxyapatite that is chemically and structurally similar to the mineral phase of native bone, scaffolds can achieve better biological activities and have spontaneous induction of bone formation [12].

Among the numerous 3D lattice-based geometries, the diamond unit cell is a promising material for orthopedic applications [13,14,15,16]. It is an isotropic geometry where each connection node of the struts is tetrahedron-like, surrounded by four other connection nodes [17]. It has been considered to possess self-supporting properties, enhancing the capability of the additive manufacturing (AM) process to fabricate the cellular lattice structures with wide ranges of volume fraction and at a large scale without any deformation [18]. A number of works have studied this porous unit in terms of its manufacturability [19,20], mechanical properties by analytical solutions [16] or numerical simulation [5], biological activity [13]. In addition, the mechanical properties and interconnected porosity of diamond lattice are close to those of trabecular bone [13].

However, they are not without drawbacks, as lattice structures (including diamond structure) employing a unit cell with straight beam-like struts and sharp turns are found to be anti-biomorphic because they give rise to aggregates of cells that have a negative effect on homogeneous migration and proliferation [10,21]. They also have been shown to form stress concentrations, resulting in local defects and thus decreasing the fatigue life. F.Brenne et al. [22] studied the mechanical behavior of open cellular structures under monotonic and cyclic loading, indicating that local strains contributed to failure at an early stage. Besides this, the junctions where the struts connect to each other are considered to be thicker than expected due to the unmelted or semi-melted powders on the surface with straight edges and sharp turns [23]. A promising mathematic approach, triply periodic minimal surfaces (TPMS), has been used for designing lattice structures with smooth surfaces due to its capacity to produce functionally gradient and hierarchical porous structures [24,25,26]. TPMS architectures, including gyroid and diamond, own more homogeneous stress distribution and hence higher stiffness than the cubic lattice with straight beam-like struts and sharp turns [8,24]. Taking the topological optimization design into account, the CAD-based approach can be more finely tuned for the improvement of the structures.

At the same time, these lattice structures with high inner complexity are still facing the substantial challenges of fabrication technology. Conventional processing technologies used for scaffold production include gas-foaming, salt leaching, and phase-separation followed by freeze-drying, which contained relatively rough geometries and a lack of structure controllability [27]. A number of open-cell porous structures with a homogeneous unit, such as polyhedral units [28,29] or TPMS units [30], have been successfully fabricated by these AM techniques and extensively developed. In contrast, selective laser melting (SLM) or laser powder bed fusion (LPBF), as an additive manufacturing process, is more suitable for manufacturing metallic scaffolds with cellular lattice structures because of its high precision and excellent performance [5,10,31]. SLM based on powder bed has been used to form the finished part directly layer-by-layer without any aid of tools and molds. It has the capacity to build the inner complex shape of metal parts with a high density, above 99.5% [32]. Additionally, SLM becomes a superior candidate for producing tissue engineering scaffolds by accurately governing parameters such as pore size, porosity, and interconnectivity [5].

In this regard, the aim of this study was to investigate the regular scaffolds in different volume fractions for mechanical properties requirements of osteointegration and evaluate the effect of topological optimization on the structural performances. Mechanical properteies, as one of the key factors essential for biomaterials, have been focused on here, and this attempt is expected to meet a part of the biological requirements. Firstly, internal pore architectures using diamond unit cells were designed by smooth optimized surfaces at unit nodes with several parameters accurately controlling porosity and pore size. Secondly, Ti6Al4V alloy samples with volume fractions ranging from 1.28 to 18.6% were fabricated using SLM system, followed by uniaxial compression experiments to measure the constitutive properties of SLM Ti6Al4V lattice structures, including compressive strength and elastic modulus to match the levels of human bones. Finally, the performance of diamond structures were demonstrated to be modified with surface optimization, resulting from the stress concentration reduction and the difference in the failure mechanism.

## 2. Materials and Methods

### 2.1. Design of Cellular Lattice Structures

**Figure 1:**
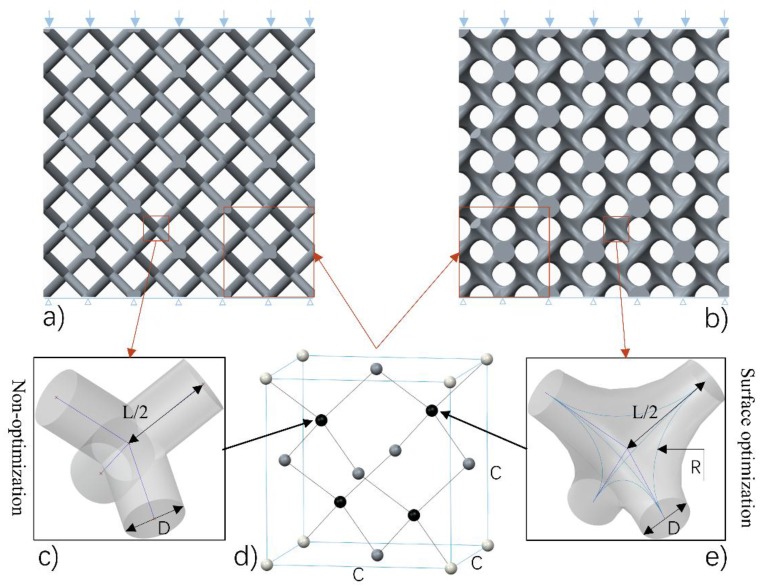
The experimental conditions: (**a**) sample 0600 without optimization, (**b**) sample 0616 with surface optimization at nodes (where ‘06’ denotes D = 0.6 mm, ‘16’ denotes R = 1.6 mm), (**c**) a node without optimization, (**d**) one of the unit cells used in the diamond-type 3 × 3 × 3 cellular structures, (**e**) a node with optimized surface.

The diamond lattice structures studied in this paper were generated by means of a CAD-based approach. Figure 1 shows the design method and process of the diamond lattice structure. As Figure 1d shows, a diamond unit cell that has fourteen nodes and sixteen equal edges is an isotropic geometry [4]. For a unit cell, the length of each strut between two nodes (L, and size of the unit cell (C) are related to each other by using the formula $L=\frac{\sqrt{3}}{4}c$. The angle (θ) between strut and the horizontal plane equals 35°16′, which is a safe degree for avoiding deformation during the SLM process, resulting in a feature of being self-supporting. Table 1 shows the geometrical parameters of the diamond lattice structures studied in this article.

In the unit cell architecture, each optimized node is defined by rod diameter (D) and optimized radius (R). Combined with unit cell size, parameters including C, D, R are introduced as the basic elements that can accurately control pore size and volume fraction of this interconnected network. Compared with the straight beam-like nodes (Figure 1c), optimized nodes have smooth surfaces whose curvature is regulated by the value of optimized radius (Figure 1e), which is considered to own better manufacturability for SLM. Then, they are assembled into the diamond lattice (Figure 1d), forming the finished unit cell architecture.

A final architecture can be carried out by a periodic array of the unit cell along with three mutually perpendicular directions. Figure 1a,b depict diamond-type 3 × 3 × 3 cellular structures, sample 0600 with straight beam-like struts and sample 0616 with optimized surface at its unit nodes, respectively, where ‘06’ denotes D = 0.6 mm, ‘16’ denotes R = 1.6 mm, and other numbers (for example ‘1008’) are similar. In this paper, to better study the performance of diamond structures, the value of the unit cell size is set to be 5.5 mm to provide the ability for SLM manufacturing of cellular lattice structures with a wide range of volume fractions [19]. Dimensions of the test samples are fixed at 16.5 × 16.5 × 16.5 ${\mathrm{mm}}^{3}$, because 3 × 3 × 3 cell types are a compromise, combining economy with accuracy, as Smith et al. [33] studied cell size effect and stated that many properties could be well predicted with lower amounts of unit cells.

**Figure 2:**
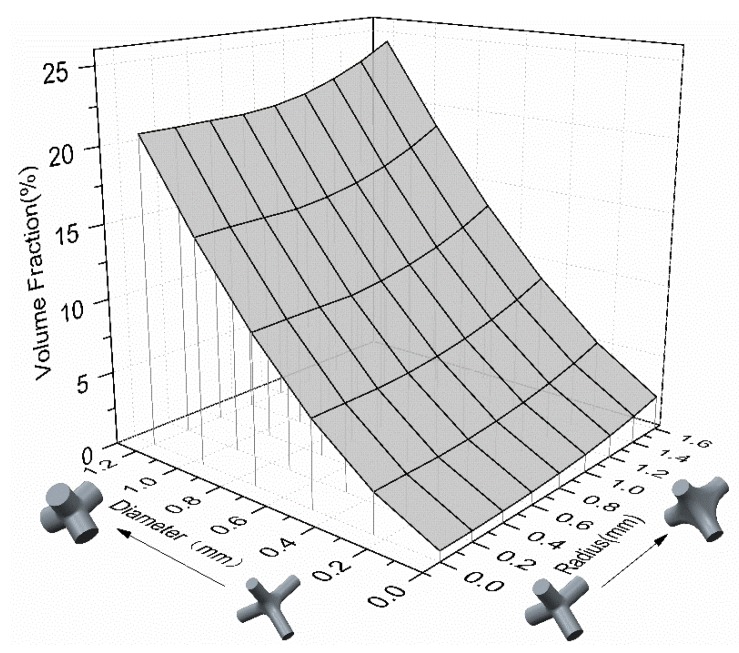
Value of volume fraction determined by rod diameter and optimized-radius.

Based on this design approach, different combinations of pore sizes, volume fraction values and mechanical properties can be obtained for specific application requirements by modifying the parameters above. The volume fraction, as one of the key factors controlling the mechanical properties of porous parts, is determined by rod diameter, optimized radius and unit cell size. As shown in Figure 2, when the unit size is set as a constant, increasing the rod diameter is an effective way to achieve the rapid growth of volume fraction, in order to get the desired mechanical properties, while an optimized radius that contributes little to the increase of volume fraction may play a role to improve and adjust the stiffness and strength of diamond structures.

### 2.2. Materials

**Figure 3:**
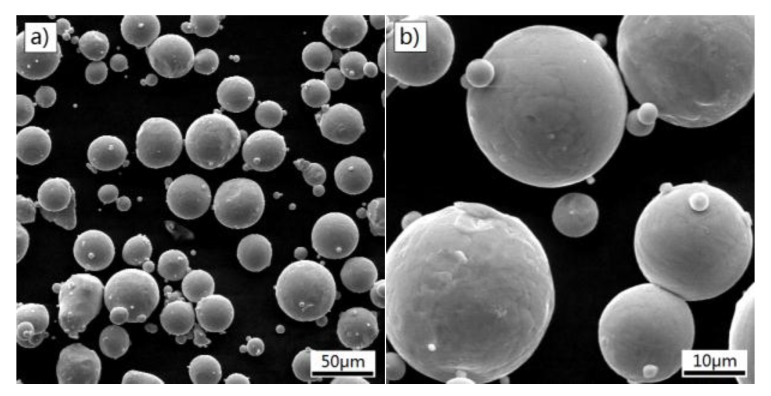
SEM micrographs of the Ti6Al4V powder, (**a**) ×500 and (**b**) ×2000.

Titanium alloy is an important metal for medical devices because of its excellent osseointegration, superior corrosion resistance and favorable mechanical properties. Thus, cellular lattice structures were made from Ti6Al4V powder that was produced by a gas atomization process. As shown in Figure 3, SEM images of Ti6Al4V powder exhibit a feature of a nearly spherical shape and smooth surfaces, indicating a good flowability. The particle size of the powder is in the range of 3–50 μm. The chemical composition is listed in Table 2. The powder has a low content of impurities (oxygen, hydrogen and nitrogen), which might avoid negative effect of embrittlement [34].

### 2.3. Fabrication by SLM

**Figure 4:**
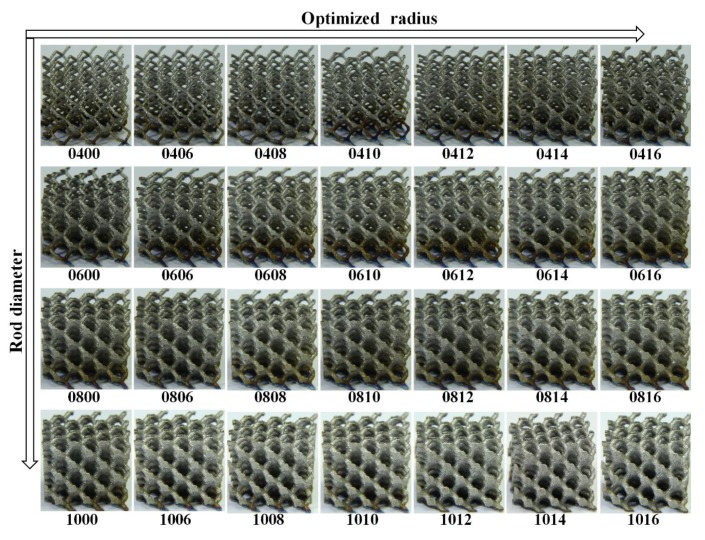
The formed samples by selective laser melting with a dimension of 16.5 × 16.5 × 16.5 ${\mathrm{mm}}^{3}$ (where ‘10′ denotes D = 1.0 mm, ‘16′ denotes R = 1.6 mm, and other numbers (for example, ‘1008′) are similar).

**Figure 5:**
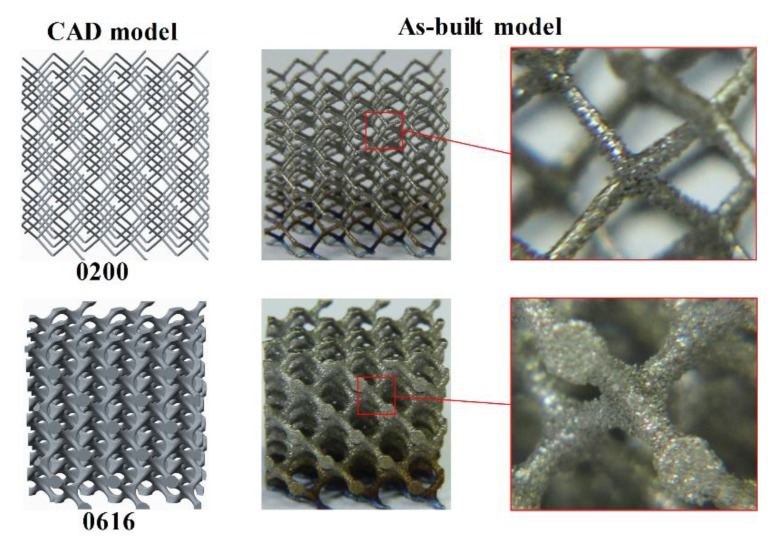
Comparison between CAD models and as-built samples.

Cellular lattice structures with rod diameter from 0.2 to 1.0 mm and optimized radius from 0 to 1.6 mm were manufactured by a SLM machine (EOSINT-M280, EOS GmbH, Munich, Germany) equipped with a 200 W CW ytterbium fiber laser. The diameter of the laser beam focused on the powder bed is 0.1 mm. The laser melting process occurs in a protective argon atmosphere with $O_{2}$ content less than 0.1 vol.%, and the processing parameters were set up as follows: the laser power was 170 W; the track length of the laser beam was 5 mm; the hatch spacing was 75 μm; the scanning speed was 1250 mm/s; the beam offset was 0.015 mm; and the layer thickness was 30 μm. Using these parameters, the density of bulk Ti6Al4V was measured as $4.42\;g/{\mathrm{cm}}^{3}\left(\geq 99.5\%\right)$ by the Archimedes method, tensile strength was 930 MPa and modulus of elasticity was 116 GPa after a post processing of heat treatment. There were 33 samples in a uniform dimension of $16.5\times 16.5\times 16.5{\;\mathrm{mm}}^{3}$ built in one batch to reduce deviation during fabrication. When the SLM process was finished, the proposed samples were removed from the base plate via wire electrical discharge machining (wire-EDM); Figure 4 shows the samples for compressive test. Figure 5 exhibits the comparison between CAD models and as-built samples that was observed under an optical microscope, revealing a good consistency. Even the sample 0200 with the volume fraction of 1.28% (theoretical value) and rod diameter of 0.2 mm, which is considered to be the most difficult to form, has been manufactured successfully with no defects or broken cells, confirming the capability of SLM to fabricate these cellular lattice structures. For the compressive test, the as-built samples were subjected to a post processing of heat treatment (annealing for 3 h at 650 °C) in an argon gas atmosphere with subsequent furnace cooling for thermal stress releasing.

### 2.4. Test Details

Uniaxial compressive tests of the lattice structures were conducted by using a universal testing machine equipped with a 100 KN load cell. Most of the samples subjected to load were parallel to the building direction, except for those samples signed “XY” whose direction of loading was parallel to the layer. The compressive tests were carried out at a displacement rate of 2 mm/min in a continuous process without intermittence, accompanied by a video camera with a resolution of 10 megapixels and a frame rate of 29 fps for continuously recording the deformation of each sample. The camera was equipped with a macro lens and a spotlight, ensuring the proper illumination necessary for digital image correlation (DIC) analyses. Then, frames correlated with strain were extracted from these videos, providing information about the failure behavior of the lattice structures. In order to compare the uniaxial test results, the strain was calculated by dividing the displacement by sample height of 16.5 mm, while the stress was computed from the load and apparent cross-sectional area of 16.5 × 16.5 ${\mathrm{mm}}^{2}$. Elastic modulus (E) was considered as the slope of linear fits of the stress-strain curve. And stress at the first peak of the experimental data was regarded as ultimate stress (σ).

## 3. Results and Discussion

### 3.1. Analysis of Mechanical Properties 

In general, comparisons for the same topology of micro-lattice are made according to the volume fraction or relative density which is calculated by dividing the density of micro-lattice block (ρ) by that of parent material ($\rho_{0}$). So the volume fraction (*Vf*), porosity (*P*), were calculated as:

(1) $$Vf=1-P=\rho /\rho_{0}\times 100\%$$

In addition, in order to evaluate the effect of optimized radius on mechanical properties of diamond lattice structure, relative modulus ($E^{*}$) and relative ultimate stress ($\sigma^{*}$) are adopted to assess its material efficiency, namely:

(2) $$E^{*}=\frac{E}{Vf}$$

(3) $$\sigma^{*}=\frac{\sigma }{Vf}$$

**Figure 6:**
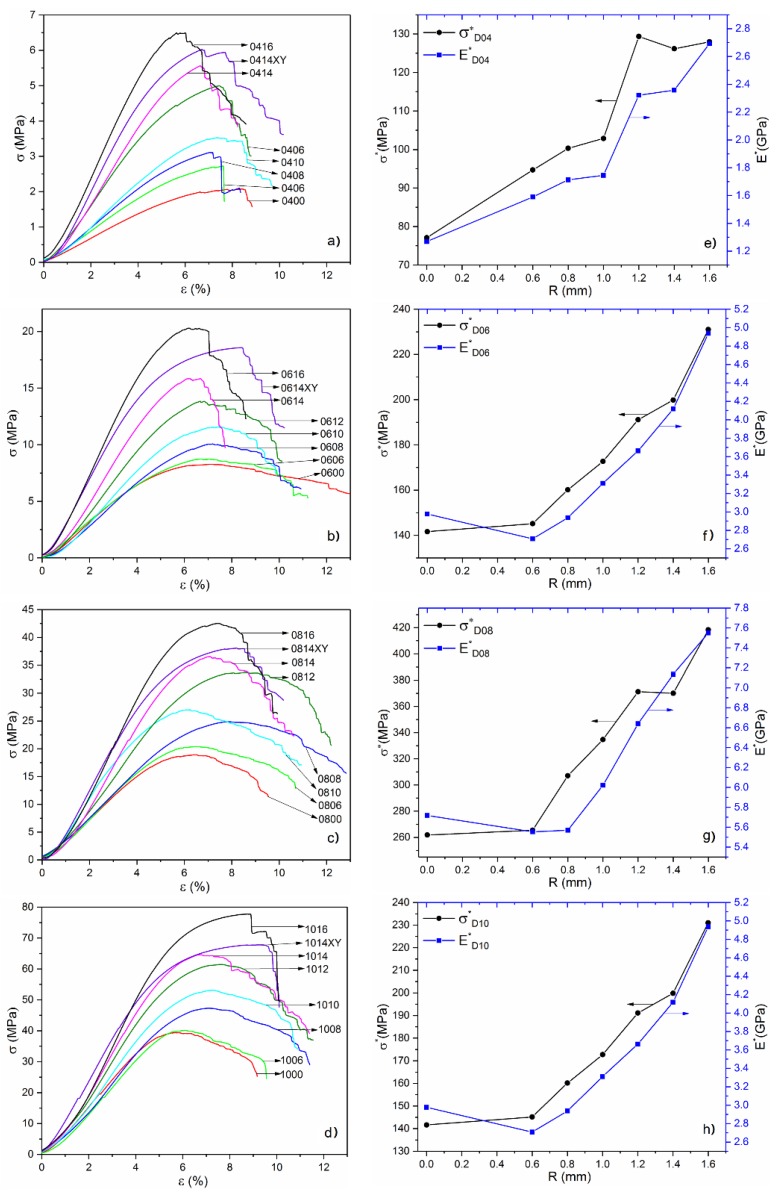
(**a**–**d**) The stress–strain curves; (**e**–**h**) the corresponding relative ultimate stress $\sigma^{*}$ and relative modulus $E^{*}$.

Stress–strain curves of samples with rod diameter of 0.4, 0.6, 0.8, 1 (mm) are shown in Figure 6a–d, respectively. These stress–strain responses are characterized by a nearly linear region followed by yield at a plastic region that maintained constant stress in a longer strain range due to the influence of plasticity enhancement with the increase of the rod radius. The corresponding $\sigma^{*}$ and $E^{*}$ values with optimized radiuses from 0.6 mm to 1.6 mm are depicted in Figure 6e–h. It is observed that the relative ultimate stress and relative modulus of the samples are distinctly linearly increasing with the increase of optimized radius, whatever the sizes of the rod diameter are, indicating that the surface optimization at nodes can improve the mechanical properties of diamond lattice structure and enhance the performance efficiency of this cellular materials. The introduction of surface optimization was considered to produce more uniform transmission of external force, leading to the reduction of stress concentration and the change of stress distribution on each strut cell. Therefore, it can be speculated that the mechanical properties of other lattice structures can be further improved by taking measure such as, as a simple way, the surface optimization at nodes.

On the other hand, an important concept to note is the anisotropy. By comparing the data obtained from different loading direction shown in Table 2, it is obvious that both the elastic modulus and ultimate strength of samples perpendicular to the building direction (signed “XY”) are higher than the parallel ones. The anisotropy of mechanical properties that are commonly found in SLM parts can be attributed to the oriented growth process along the direction of substrate layer-by-layer [35].

It is well known that the higher a volume fraction is generated, the better mechanical properties of porous materials. In order to derive a predictable relationship between mechanical properties and volume fraction, a significant theoretical model of Gibson–Ashby was used as following [10,36]:

(4) $$\frac{E}{E_{0}}=C_{1}{\left(\frac{\rho }{\rho_{0}}\right)}^{n_{1}}$$

(5) $$\frac{\sigma }{\sigma_{0}}=C_{2}{\left(\frac{\rho }{\rho_{0}}\right)}^{n_{2}}$$

where $E_{0}$, $\rho_{0}$ and $\sigma_{0}$ are the elastic modulus, density and strength of fully dense solid materials, and *E*, ρ and σ are the apparent modulus, density and strength of studied lattice samples, respectively. The exponents $n_{1}$ and $n_{2}$ are the density factors. According to the previous studies, $n_{1}$ and $n_{2}$ were calculated to be 2 and 1.5, respectively, by theoretical computational models [16] and by experimental approach [14,36]. The Gibson–Ashby prefactor $C_{1}$ and $C_{2}$ are influenced by the pore shape and size, the value of these could be changed by the introduction of optimized radius. Using experimental data can yield the corresponding value, expressed as

(6) $$\Rightarrow C_{1}=\left(\frac{E}{E_{0}}\right)/{\left(\frac{\rho }{\rho_{0}}\right)}^{2}$$

(7) $$\Rightarrow C_{2}=\left(\frac{\sigma }{\sigma_{0}}\right)/{\left(\frac{\rho }{\rho_{0}}\right)}^{1.5}$$

**Figure 7:**
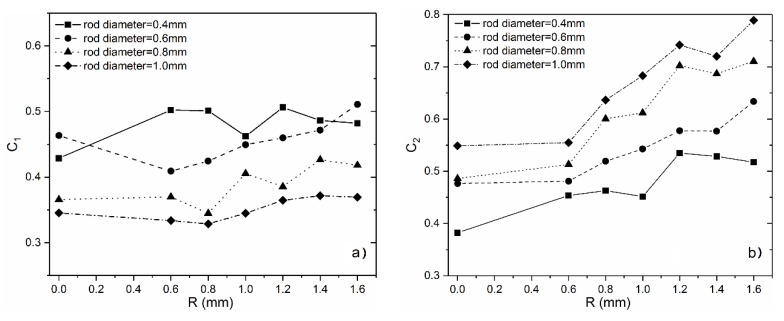
Values of $C_{1}$ and $C_{2}$ with the increase of optimized-radius.

According to the experimental values in Table 3, the corresponding constant $C_{1}$ and $C_{2}$ values can be calculated by the Equations (6) and (7), as shown in Figure 7. It is observed that, with the increase of the optimized radius, $C_{1}$ keeps almost constant or has increased by a small margin, while $C_{2}$ has increased significantly, which indicates that the surface optimization at the nodes contributes more to the improvement of strength than elastic modulus. In comparison, although relative elastic modulus and the relative strength in previous section have been improved simultaneously. While in the view of the Gibson–Ashby model, only strength is enhanced in fact. That is to say, there is a difference in influence of optimized surface at lattice nodes on their elastic modulus and the compressive strength, which may provide a way to adjusting the relationship between them by varying the curvature of optimized surface.

### 3.2. Bone Modulus Matching

Table 3 lists the performance data of all tested samples. The diamond cellular lattice structures studied in this paper have an interconnected high porosity of 81–97% that is considered suitable for tissue ingrowth and vascularization [13]. These three-dimensional structures with high interconnected porosity provide biological anchorage for the ingrowth of mineralized tissue into the pore space, enhancing the fixation of the implant in the surrounding bone as well as improving its long-term stability [10]. As Figure 2 shows a wide range of the porosity with controllability, the studied lattice structures provide great promise for creating biological alternatives for implants, in which the interconnected porosity plays a vital role in cell seeding, nutrients transfer to stimulate bone formation. 

Some of the literatures cataloged the properties of cortical bone and trabecular bone from multiple locations within the human skeleton, as summarized in Table 4. 

One of the most striking features of bone’s properties is the huge variation in modulus and strength [41]. Comparison between Table 3 and Table 4 shows that the elastic modulus of diamond structures and human bone are in a similar range, which is a significant conclusion for the application of the lattice titanium structures built by SLM as biomaterials for bone replacement. As a consequence, the diamond structure reveals properties comparable with trabecular bone, which can minimize the stress-shielding effect and increase the longevity of implants. What needs to be pointed out is that the listed values in Table 4 should only represent the dimensions of bone’s properties, because the properties of bone depend on numerous factors, such as anatomical site, the age and sex of the bone tissue, methods of storage and testing conditions [13]. Thus, more requirements should be given to scaffold for replacing natural tissues, for example, by constructing gradient or heterogeneous structures for satisfying multi-scale structures in human bone. The performance of diamond lattice shown in Table 3 is expected to provide a reference in the future study.

**Figure 8:**
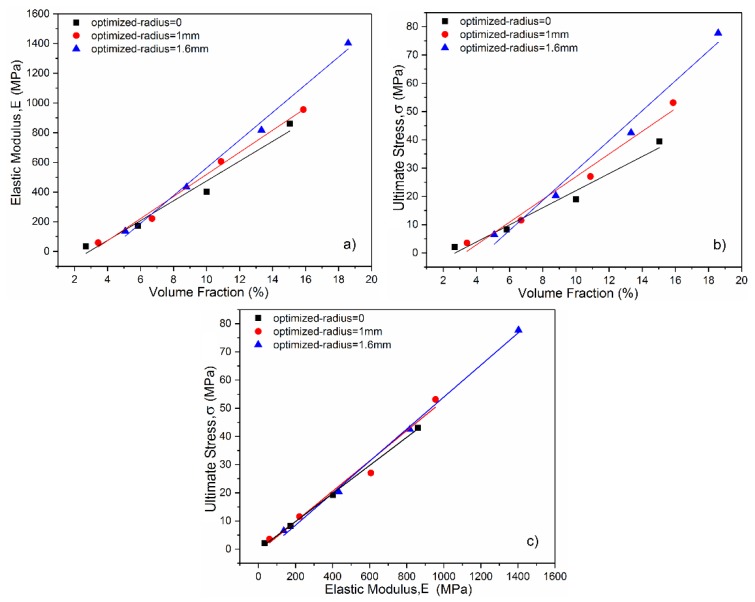
Mechanical properties of the diamond structures with different optimized-radius: (**a**) Elastic modulus; (**b**) ultimate stress; (**c**) the relationship between elastic modulus and compression strength.

Figure 8a,b show plots of ultimate stress versus volume fraction values and the elastic modulus versus volume fraction values, respectively. It is clear that strength and modulus increase linearly with the increase of density, and the linear coefficients of optimized surface structures are higher than the ones of non-optimization. It was indicated that the optimized radius played a role in regulating the relationship between density and mechanical properties including the strength and modulus. This regulation controlled by the value of optimized radius may extend the range of mechanical properties of the diamond structure without changing volume fraction and permeability. In addition, the smooth surface inner complex shape contributes to removing the unmelted or semi-melted powders and enhancing surface finish by post-processing such as magnetic fluid-assisted polishing and electrochemical polishing, as these micro powders on the implant surface could trigger inflammation.

A linear relationship was found between the elastic modulus and strength in Figure 8c, which is similar to the properties of bone stated in the previous article [41]. Figure 8c also shows that the compression strength of the optimized structures is superior to that of non-optimized structures under the same condition of elastic modulus, increasing fatigue endurance for long-term application in the human body. Moreover, it is possible to accurately predict and even tailor the mechanical properties of the diamond lattices by governing the volume fraction and adjusting the curvature of node surface, because the relationship between the mechanical properties and volume fraction has been exactly established.

### 3.3. Deformation Behavior

**Figure 9:**
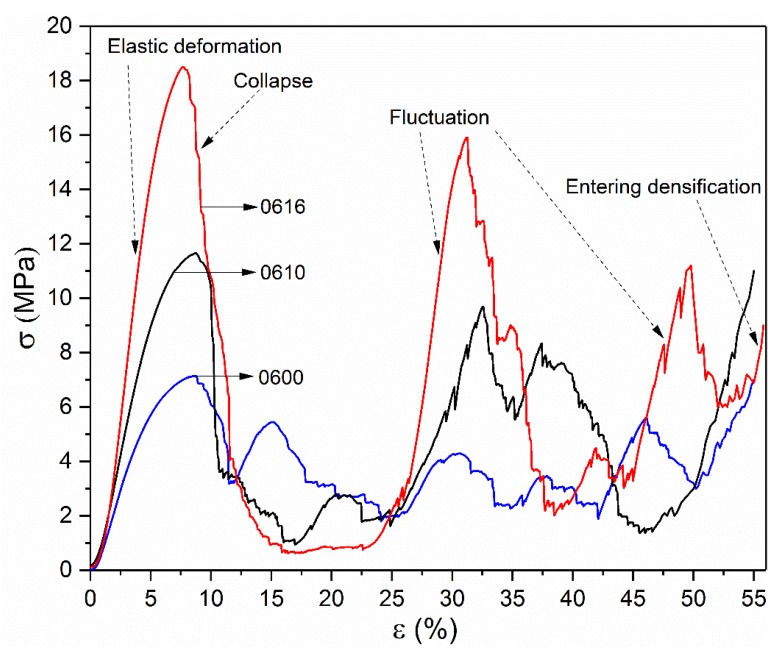
Compressive stress–strain curve of 0600, 0610 and 0616 samples.

The compressive force-deformation behaviors of 0600, 0610 and 0616 samples are plotted in Figure 9. All of them experienced four distinct regions: a region of elastic deformation up to the first peak value, followed by a collapse in which most of the strength is lost, then a region of fluctuation in stress, and a densification region characterized by a rapid increase in stress, as shown in Figure 9. These regions are in good agreement with that observed in the previous investigations [14,42].

The stress–strain curve of the 0600 sample shows a significant plateau of stress in the fluctuant region, indicating a typical characteristic of brittle deformation behavior [5]. In comparison, with the increase of the optimized radius, the stiffness and strength have been greatly enhanced, whereas the amplitude in the fluctuant region becomes higher gradually. It should be noted that, in the region of collapse, all of these curves bore high maximum stress before a steep drop that occurred at strains of 8.74%, 8.78%, and 7.71% respectively. The bigger optimized radius was used, the steeper and deeper stress drop occurred, resulting in a long-term low stress in 0616. These differences, on one hand, can be attributed to the effect of the increase of volume fraction from optimized surface at nodes. On the other hand, the more important reason is that the distribution of stress in the unit structure has been changed by the smooth surface, resulting in the release of stress concentration of unit cell and the increase of stability of the lattice structure. Moreover, strain–stress curves show that higher load bearing capacity can be expected when stress concentration has been released, leading to the mechanism of the shearing crack for sample 0616, as shown in Table 5.

Table 5 shows the video frames of samples in the complete stress–strain process. Observing their deformation behavior, each fracture occurred as a random event in a non-uniform way on the local scale of each sample, but the overall deformation behavior was determined by the structure itself and parent material. In addition, the change of stress distribution has a significant influence on the crushing behavior of these structures. The impact of the control parameter of optimized radius on the mechanical properties can be revealed for different deformation patterns.

**Figure 10:**
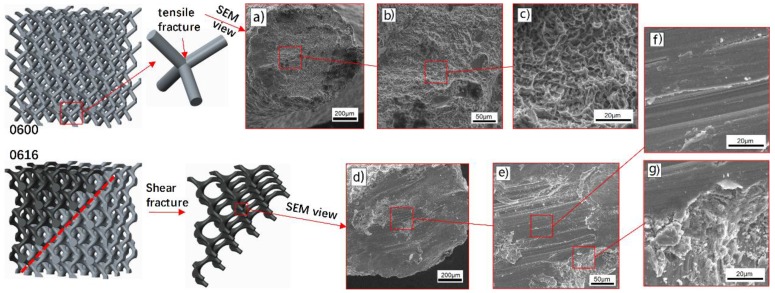
SEM morphologies of fracture surface: (**a**–**c**) tensile yield at node with ductile dimpling for sample 0600, (**d**–**g**) shear rupture at rod for sample 0616.

Failure mechanism of sample 0600 without optimized surface at nodes was accompanied by a layer-by-layer deformation from the bottom up. Due to the effect of the successive layers of collapse, 0600 has given a periodic stress fluctuation curve with a small amplitude until the densification region, indicating that the stretch dominated deformation behavior played a key role in the deformation mechanism of such structures. Furthermore, fracture occurred almost exclusively at the lattice nodes, and then rod-shaped struts separated from junction nodes were observed during the compression process owing to the buckling at the junction. SEM morphologies of this fracture surfaces shown in Figure 10 provide a fracture mechanism of tensile yield with ductile dimpling.

The in-situ images of 0610 show that, when the strain went to 10–15%, a diagonal (45°) shear band was observed; at the same time, the lowest unit cells were also destroyed. Deformation was accompanied by a layer-by-layer buckling and a diagonal (45°) shear band simultaneously, leading to a slight fluctuation in the lowest stress region, which is similar to the secondary peak in the 0600 stress–strain curve.

In contrast, testing sample 0616, destruction was mainly presented in the pattern of a diagonal (45°) shear band, whereas upward-layer failure pattern was not observed. The SEM micrographs of sample 0616 in Figure 10 provide evidence that the fracture micro-mechanisms are quite different from that of 0600. They show a typical shear rupture along the slip plane, ensuring the assumption mentioned above that the loading stress has been transferred from the node to the rod center under the influence of optimized surface. This transference gives rise in the reduction of stress concentration, resulting in the improvement of mechanical properties.

Nevertheless, effected by the collective failure on the diagonal (45°) shear band, the curve of 0616 showed a more severe collapse, in which 95% of the structure’s strength was lost, following a long-term process of low-stress and a secondary linear elasticity behavior. The occurrence of this secondary linear elasticity is principally because other structures outside the shear band are involved in the deformation after the contacts of the struts in the shear band. As shown in Table 6, failure mechanism of sample 0616 was in good agreement with diamond structures as in previous article [5], but their optimum conditions were different. Both sample 0616 and structure of [5] showed the same deformation pattern of bending dominated under different condition in which non-optimized structure had a volume fraction of 22%, while optimized one had 8.97%. Similarly, an entirely different deformation pattern was found in the same structure, the main difference between 0600 and [5] is the volume fraction. Comparison in Table 6 indicates that the deformation pattern is determined not only by the unit cell type consisting of porous structures [43] but also by the volume fraction of the structure and the stress distribution of the nodes. According to Deshpande et al. [44], the deformation behavior of the diamond structure is bending dominated, but the sample 0600 exhibits a tendency of stretch dominated in the pattern of a layer-by-layer failure, which demonstrated the effect of volume fraction on deformation pattern.

## 4. Conclusions

In this study, a diamond unit cell with an optimized surface at nodes was designed in order to improve and adjust its mechanical properties. The diamond cellular lattice structures with a wide range of volume fractions from 1.28% to 18.6%, having a unit cell size (5.5 mm), were manufactured by the SLM using the Ti6Al4V alloy, showing the great flexibility of this design approach and its suitability for matching the properties of porous human bone. The effects of the node optimization on the mechanical properties and deformation behavior under compressive test were investigated. The major findings of this research are summarized as follows.

1. Mechanical properties including strength and modulus increase with the increase of optimized radius at a fixed volume fraction, indicating that the stress concentration at lattice nodes have been released by the introduction of surface optimization;
2. A minitype database that correlates porosity levels to mechanical properties has been established. The compressive strength and elastic modulus of diamond lattice structures are in the range of 2–78 MPa and 34–1403 MPa, respectively, which are comparable with trabecular bone;
3. By optimizing the nodes with different curvature in each unit cell, the relationship between compressive strength and elastic modulus can be regulated, enabling the cellular structures more suitable for bone implants implication;
4. The change of stress distribution stemming from the optimized surface at nodes has a significant influence on the deformation behavior of these structures. With the analysis of in-situ images and SEM, it is revealed that the improvement of mechanical properties was mainly due to the transition of the deformation behavior from upward-layer collapse in non-optimization structures to diagonal (45°) shear band in optimized ones.

Although diamond structures fabricated by SLM show a good potential in terms of mechanical tailoring for bone scaffolds, there are some defects to be resolved at the present stage. It is a time-consuming and tedious manual process for CAD-based lattice structures, especially for more complex functionally graded scaffolds. In order to better mimic host tissue, scaffolds with hybridizing structures are needed to satisfy the biological requirements for the better regeneration of bone tissue. The database of mechanical properties studied in this article can be a reference for bone scaffold applications. Besides this, effected by the limitations in the diameter of the laser beam and powder particle size, the geometric feature size of the studied scaffolds is still too large to match that of human bone. With the development of SLM manufacturing in the future work, more accurate parts to be fabricated will be desirable. There still is a long distance to application due to some other biological factors which are not strictly addressed in this work. Other measures such as spraying hydroxyapatite to improve the biological activity and testing the permeability are supposed to be taken in the future work.

## Figures and Tables

**Table 1:** Geometrical parameters of the diamond lattice structures.

Parameters	C (mm)	D (mm)	R (mm)	L (mm)	θ (mm)
CAD size	5.5	0.2–1.0 (interval 0.2)	0–1.6 (interval 0.2)	$\sqrt{3}c/4$	35°16′

**Table 2:** Chemical composition of Ti6Al4V (wt.%).

Ti6Al4V	Ti	Al	V	O	N	C	H	Fe
Wt.%	(balance)	5.5–6.75	3.5–4.5	<0.2	<0.05	<0.08	<0.015	<3

**Table 3:** Characteristics of cell structure and mechanical properties of open cellular Ti6Al4V samples.

Samples	Rod Diameter, D (mm)	Optimized-Radius, R (mm)	Volume Fraction, *Vf* (%)	Modulus, E (MPa)	Ultimate Stress, σ (MPa)
0200	0.2	0	1.28	--	--
0400	0.4	0	2.69	34.12	2.07
0406		0.6	2.88	45.75	2.72
0408		0.8	3.11	53.17	3.12
0410		1	3.43	59.86	3.53
0412		1.2	3.86	89.68	5.00
0414		1.4	4.41	103.97	5.56
0416XY		1.4	4.41	122.34	6.03
0416		1.6	5.08	136.66	6.50
0600	0.6	0	5.84	173.80	8.27
0606		0.6	6.02	162.97	8.74
0608		0.8	6.29	184.73	10.07
0610		1	6.7	221.64	11.56
0612		1.2	7.24	265.25	13.84
0614		1.4	7.94	326.79	15.86
0614XY		1.4	7.94	370.69	18.58
0616		1.6	8.79	433.83	20.30
0800	0.8	0	10	402.75	18.92
0806		0.6	10.14	418.52	20.39
0808		0.8	10.43	412.53	24.87
0810		1	10.88	606.42	27.02
0812		1.2	11.51	561.95	33.73
0814		1.4	12.33	712.83	36.56
0814XY		1.4	12.33	748.58	38.07
0816		1.6	13.33	817.10	42.52
1000	1.0	0	15.04	860.01	39.38
1006		0.6	15.13	840.26	40.16
1008		0.8	15.4	857.61	47.29
1010		1	15.87	955.56	53.12
1012		1.2	16.55	1099.06	61.45
1014		1.4	17.45	1245.33	64.59
1014XY		1.4	17.45	1260.84	67.79
1016		1.6	18.58	1402.69	77.73

Note: All of the samples have a unit size of 5.5 mm. The volume fraction values are theoretical based on designed models. ‘XY’ denotes the loading direction is perpendicular to the building direction.

**Table 4:** Mechanical properties of human cortical and trabecular bones.

Material		E (MPa)	Direction and Type of Load	$\sigma_{\max}$ (MPa)	Reference
Cortical bone	Mid-femoral	Mean:17(×103)	Longitudinal	Mean: 193	[37]
		Mean:11.5(×103)	Transverse	Mean: 33	
	--	14.1–27.6(×103)	Longitudinal	219 ± 26	[38]
			Transverse	153 ± 20	
Trabecular bone	Proximal femoral	Mean:441	Longitudinal	Mean: 6.8	[37]
	--	100–400	--	1.5–9.3	[38]
	Femoral bone	65.7–873 (mean: 315)	--	0.8–12.7 (mean: 6)	[39]
	Proximal tibia	4–430	Longitudinal	1–13	[40]

**Table 5:** Video frames of samples at several regions during compression.

Num	$\varepsilon_{latt}$ (%)					
	5	10	15	20	30	40
0600	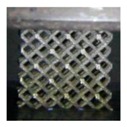	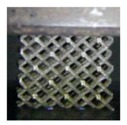	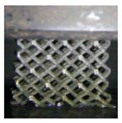	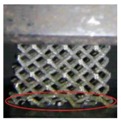	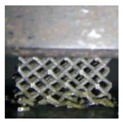	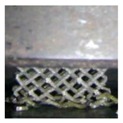
0610	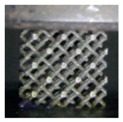	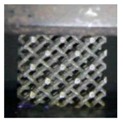	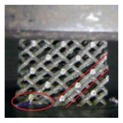	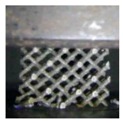	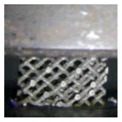	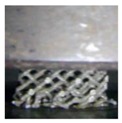
0616	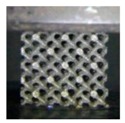	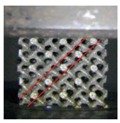	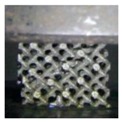	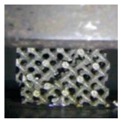	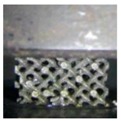	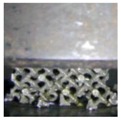

**Table 6:** Failure mechanism comparison with previous article.

Structures	Optimization	Volume Fraction, (%)	Deformation Modes		Deformation Pattern
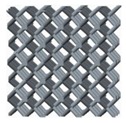	Non	5.84	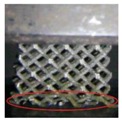	Layer-by-layer (cell row)	stretch dominated
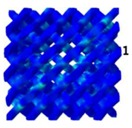	Non	22	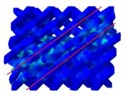	45° shear	bending dominated
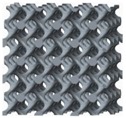	Surface optimization at nodes	8.79	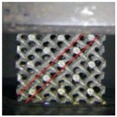	45° shear	bending dominated

^1^ Ref. [5].

## References

[1] Evans A.G., Hutchinson J.W., Fleck N., Ashby M.F., Wadley H.N.G. (2001). The topological design of multifunctional cellular metals. Prog. Mater. Sci..

[2] Olivares A.L., Marsal È., Planell J.A., Lacroix D. (2009). Finite element study of scaffold architecture design and culture conditions for tissue engineering. Biomaterials.

[3] Wang X., Xu S., Zhou S., Xu W., Leary M., Choong P., Qian M., Brandt M., Xie Y.M. (2016). Topological design and additive manufacturing of porous metals for bone scaffolds and orthopaedic implants: A review. Biomaterials.

[4] Ahmadi S.M., Campoli G., Amin Yavari S., Sajadi B., Wauthle R., Schrooten J., Weinans H., Zadpoor A.A. (2014). Mechanical behavior of regular open-cell porous biomaterials made of diamond lattice unit cells. J. Mech. Behav. Biomed..

[5] Kadkhodapour J., Montazerian H., Darabi A.C., Anaraki A.P., Ahmadi S.M., Zadpoor A.A., Schmauder S. (2015). Failure mechanisms of additively manufactured porous biomaterials: Effects of porosity and type of unit cell. J. Mech. Behav. Biomed..

[6] Cansizoglu O., Harrysson O., Cormier D., West H., Mahale T. (2008). Properties of ti–6al–4v non-stochastic lattice structures fabricated via electron beam melting. Mater. Sci. Eng. A-Struct..

[7] Cheng X.Y., Li S.J., Murr L.E., Zhang Z.B., Hao Y.L., Yang R., Medina F., Wicker R.B. (2012). Compression deformation behavior of ti-6al-4v alloy with cellular structures fabricated by electron beam melting. J. Mech. Behav. Biomed..

[8] Kapfer S.C., Hyde S.T., Mecke K., Arns C.H., Schröder-Turk G.E. (2011). Minimal surface scaffold designs for tissue engineering. Biomaterials.

[9] Zhang Z., Jones D., Yue S., Lee P.D., Jones J.R., Sutcliffe C.J., Jones E. (2013). Hierarchical tailoring of strut architecture to control permeability of additive manufactured titanium implants. Mater. Sci. Eng. C-Mater..

[10] Yan C., Hao L., Hussein A., Young P. (2015). Ti–6al–4v triply periodic minimal surface structures for bone implants fabricated via selective laser melting. J. Mech. Behav. Biomed..

[11] Melchels F.P.W., Barradas A.M.C., van Blitterswijk C.A., de Boer J., Feijen J., Grijpma D.W. (2010). Effects of the architecture of tissue engineering scaffolds on cell seeding and culturing. Acta Biomater..

[12] Ripamonti U., Roden L.C., Renton L.F. (2012). Osteoinductive hydroxyapatite-coated titanium implants. Biomaterials.

[13] Heinl P., Müller L., Körner C., Singer R.F., Müller F.A. (2008). Cellular ti-6al-4v structures with interconnected macro porosity for bone implants fabricated by selective electron beam melting. Acta Biomater..

[14] Li S.J., Xu Q.S., Wang Z., Hou W.T., Hao Y.L., Yang R., Murr L.E. (2014). Influence of cell shape on mechanical properties of ti-6al-4v meshes fabricated by electron beam melting method. Acta Biomater..

[15] Xiao L., Song W., Wang C., Liu H., Tang H., Wang J. (2015). Mechanical behavior of open-cell rhombic dodecahedron ti–6al–4v lattice structure. Mater. Sci. Eng. A-Struct..

[16] Babaee S., Jahromi B.H., Ajdari A., Nayeb-Hashemi H., Vaziri A. (2012). Mechanical properties of open-cell rhombic dodecahedron cellular structures. Acta Mater..

[17] Knorr T., Heinl P., Schwerdtfeger J., Körner C., Singer R.F., Etzold B.J.M. (2012). Process specific catalyst supports—selective electron beam melted cellular metal structures coated with microporous carbon. Chem. Eng. J..

[18] Hussein A., Hao L., Yan C., Everson R., Young P. (2013). Advanced lattice support structures for metal additive manufacturing. J. Mater. Process. Technol..

[19] Yan C., Hao L., Hussein A., Bubb S.L., Young P., Raymont D. (2014). Evaluation of light-weight alsi10mg periodic cellular lattice structures fabricated via direct metal laser sintering. J. Mater. Process. Technol..

[20] Yan C., Hao L., Hussein A., Young P., Huang J., Zhu W. (2015). Microstructure and mechanical properties of aluminium alloy cellular lattice structures manufactured by direct metal laser sintering. Mater. Sci. Eng. A-Struct..

[21] Rajagopalan S., Robb R.A. (2006). Schwarz meets schwann: Design and fabrication of biomorphic and durataxic tissue engineering scaffolds. Med. Image Anal..

[22] Brenne F., Niendorf T., Maier H.J. (2013). Additively manufactured cellular structures: Impact of microstructure and local strains on the monotonic and cyclic behavior under uniaxial and bending load. J. Mater. Process. Technol..

[23] Gümrük R., Mines R.A.W., Karadeniz S. (2013). Static mechanical behaviours of stainless steel micro-lattice structures under different loading conditions. Mater. Sci. Eng. A-Struct..

[24] Melchels F.P.W., Bertoldi K., Gabbrielli R., Velders A.H., Feijen J., Grijpma D.W. (2010). Mathematically defined tissue engineering scaffold architectures prepared by stereolithography. Biomaterials.

[25] Yang N., Tian Y., Zhang D. (2015). Novel real function based method to construct heterogeneous porous scaffolds and additive manufacturing for use in medical engineering. Med. Eng. Phys..

[26] Yang N., Du C., Wang S., Yang Y., Zhang C. (2016). Mathematically defined gradient porous materials. Mater. Lett..

[27] Yoo D.J. (2011). Porous scaffold design using the distance field and triply periodic minimal surface models. Biomaterials.

[28] Arabnejad S., Burnett Johnston R., Pura J.A., Singh B., Tanzer M., Pasini D. (2016). High-strength porous biomaterials for bone replacement: A strategy to assess the interplay between cell morphology, mechanical properties, bone ingrowth and manufacturing constraints. Acta Biomater..

[29] Egan P.F., Gonella V.C., Engensperger M., Ferguson S.J., Shea K. (2017). Computationally designed lattices with tuned properties for tissue engineering using 3d printing. PLoS ONE.

[30] Giannitelli S.M., Accoto D., Trombetta M., Rainer A. (2014). Current trends in the design of scaffolds for computer-aided tissue engineering. Acta Biomater..

[31] Zhang X.-Y., Fang G., Zhou J. (2017). Additively manufactured scaffolds for bone tissue engineering and the prediction of their mechanical behavior: A review. Materials.

[32] Thijs L., Verhaeghe F., Craeghs T., Humbeeck J.V., Kruth J.-P. (2010). A study of the micro structural evolution during selective laser melting of ti-6al-4v. Acta Mater..

[33] Smith M., Guan Z., Cantwell W.J. (2013). Finite element modelling of the compressive response of lattice structures manufactured using the selective laser melting technique. Int. J. Mech. Sci..

[34] Caiazzo F., Campanelli S.L., Cardaropoli F., Contuzzi N., Sergi V., Ludovico A.D. (2017). Manufacturing and characterization of similar to foam steel components processed through selective laser melting. Int. J. Adv. Manuf. Technol..

[35] Guan K., Wang Z., Gao M., Li X., Zeng X. (2013). Effects of processing parameters on tensile properties of selective laser melted 304 stainless steel. Mater. Des..

[36] Gibson L.J., Ashby M.F. (1997). Cellular Solids: Structure and Properties.

[37] Karageorgiou V., Kaplan D. (2005). Porosity of 3D biomaterial scaffolds and osteogenesis. Biomaterials.

[38] Alvarez K., Nakajima H. (2009). Metallic scaffolds for bone regeneration. Materials.

[39] Rohlmann A., Zilch H., Bergmann G., Kölbel R. (1980). Material properties of femoral cancellous bone in axial loading-part i: Time independent properties. Arch. Orthop. Traum. Surg..

[40] Goldstein S.A., Wilson D.L., Sonstegard D.A., Matthews L.S. (1983). The mechanical properties of human tibial trabecular bone as a function of metaphyseal location. J. Biomech..

[41] Goldstein S.A. (1987). The mechanical properties of trabecular bone: Dependence on anatomic location and function. J. Biomech..

[42] Maskery I., Aboulkhair N.T., Aremu A.O., Tuck C.J., Ashcroft I.A., Wildman R.D., Hague R.J.M. (2016). A mechanical property evaluation of graded density al-si10-mg lattice structures manufactured by selective laser melting. Mater. Sci. Eng. A-Struct..

[43] Amirkhani S., Bagheri R., Yazdi A.Z. (2012). Effect of pore geometry and loading direction on deformation mechanism of rapid prototyped scaffolds. Acta Mater..

[44] Deshpande V.S., Ashby M.F., Fleck N.A. (2001). Foam topology: Bending versus stretching dominated architectures. Acta Mater..

